# Supplementary material for: Quiescence improves Candida albicans survival of fungicidal drug exposure
Source: bioRxiv. 2026 Feb 28:2026.02.27.708532. Preprint. [Version 1] doi: 10.64898/2026.02.27.708532 (PMC12970326; doi:10.64898/2026.02.27.708532)
Supplement: Supplementary file 1 [file NIHPP2026.02.27.708532v1-supplement-1.pdf]

bioRxiv preprint doi: https://doi.org/10.64898/2026.02.27.708532; this version posted February 28, 2026. The copyright holder for this preprint (which was not certified by peer review) is the author/funder, who has granted bioRxiv a license to display the preprint in perpetuity. It is made available under a CC-BY 4.0 International license.

Supplementary tables

Table S1. *Candida albicans* strains used in this study. The provenance of each strain is indicated.

**Strain Name**	**Origin**	**Geographical Location**
SC5314	ATCC	Unknown
SC5314, pRPS10-40nm-GEMs (CytoGEMs)	Holt lab (LH4903)	Unknown
SC5314, pERG13-NLS-40nm-GEMs (NucGEMs), Nup49-mScarlet	Holt lab (LH4905)	Unknown
Sc5314, NAT-pTDH3-mNeonGreen-SFC1	Berman Lab (CB1759)	Unknown
GC75	Healthy individual; oral sample	South Africa
p75010	Bloodstream isolate	Belgium
p87	HIV+ patient; oral sample	South Africa
p37039	Bloodstream isolate	New Jersey, USA
p75016	Bloodstream isolate	Israel
p60002	Bloodstream isolate	Arizona, USA
p78042	Bloodstream isolate	Indianapolis, USA
p78048	Bloodstream isolate	Manitoba, Canada
p75063	Bloodstream isolate	France
p94015	Bloodstream isolate	Utah, USA
p34048	Bloodstream isolate	Turkey
p76055	Bloodstream isolate	Iowa, USA
12C	Vaginitis patient; oral sample	Michigan, USA
L26	Vaginitis patient; vaginal sample	Iowa, USA
p57072	Bloodstream isolate	Iowa, USA
p57055	Bloodstream isolate	Nebraska, USA
p37037	Healthy individual; oral sample	Wisconsin, USA
p76067	Bloodstream isolate	Ontario, Canada
19F	Vaginitis patient; vaginal sample	Michigan, Canada

bioRxiv preprint doi: https://doi.org/10.64898/2026.02.27.708532; this version posted February 28, 2026. The copyright holder for this preprint (which was not certified by peer review) is the author/funder, who has granted bioRxiv a license to display the preprint in perpetuity. It is made available under a CC-BY 4.0 International license.

Supplementary Figures

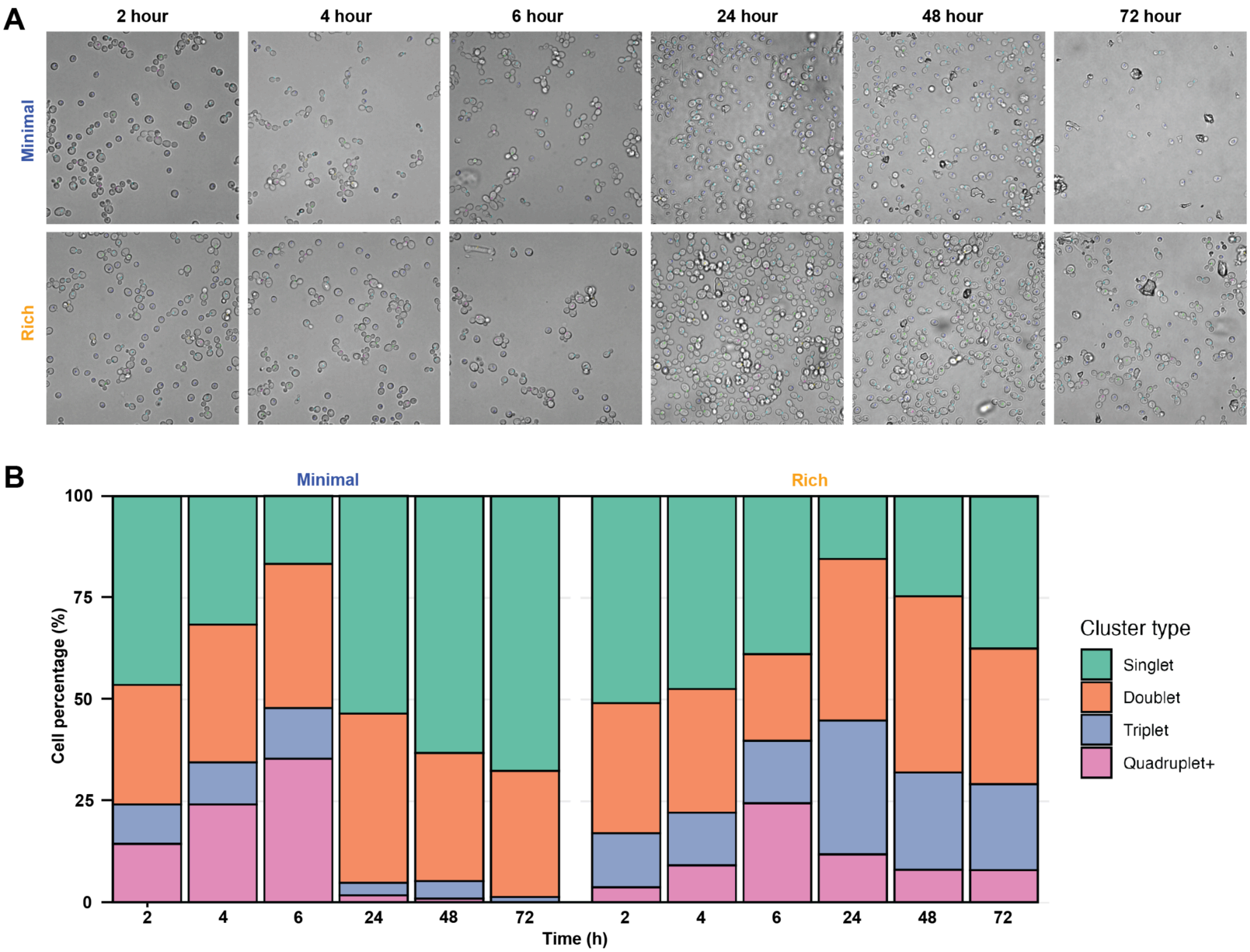

Supplementary Figure 1. Phase contrast microscopy images of rich and minimal media-grown proliferative and quiescent cells used for bud index quantification. A) Representative phase contrast microscopy images showing cells at different growth phases grown in rich and minimal media. Cells are counted and marked as 1 (singlet), 2 (doublet), 3 (triplet), 4 (quadruplet+) or 5 (ambiguous) and counted using Cell Counter plugin for ImageJ. **B)** Cell cluster type percentages change with time in cells grown in rich and minimal media. Counts quantified from Cell Counter outputs; minimum of 200 cells were counted per condition.

bioRxiv preprint doi: https://doi.org/10.64898/2026.02.27.708532; this version posted February 28, 2026. The copyright holder for this preprint (which was not certified by peer review) is the author/funder, who has granted bioRxiv a license to display the preprint in perpetuity. It is made available under a CC-BY 4.0 International license.

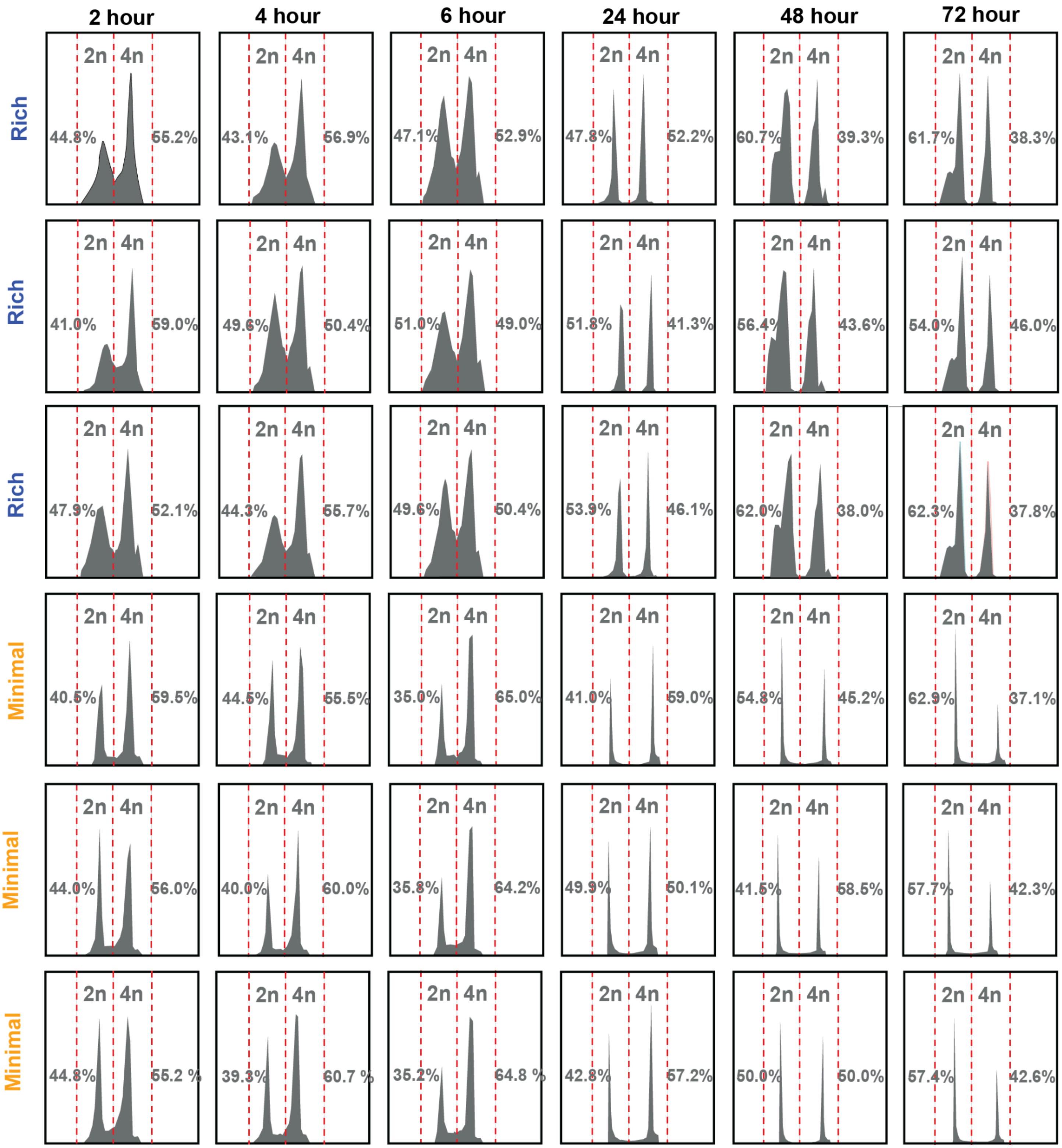

Supplementary Figure 2. DNA content analysis of proliferative and quiescent cultures in rich and minimal media. Distribution of 2n and 4n DNA peaks. N = 50,000 events for each panel.

bioRxiv preprint doi: https://doi.org/10.64898/2026.02.27.708532; this version posted February 28, 2026. The copyright holder for this preprint (which was not certified by peer review) is the author/funder, who has granted bioRxiv a license to display the preprint in perpetuity. It is made available under a CC-BY 4.0 International license.

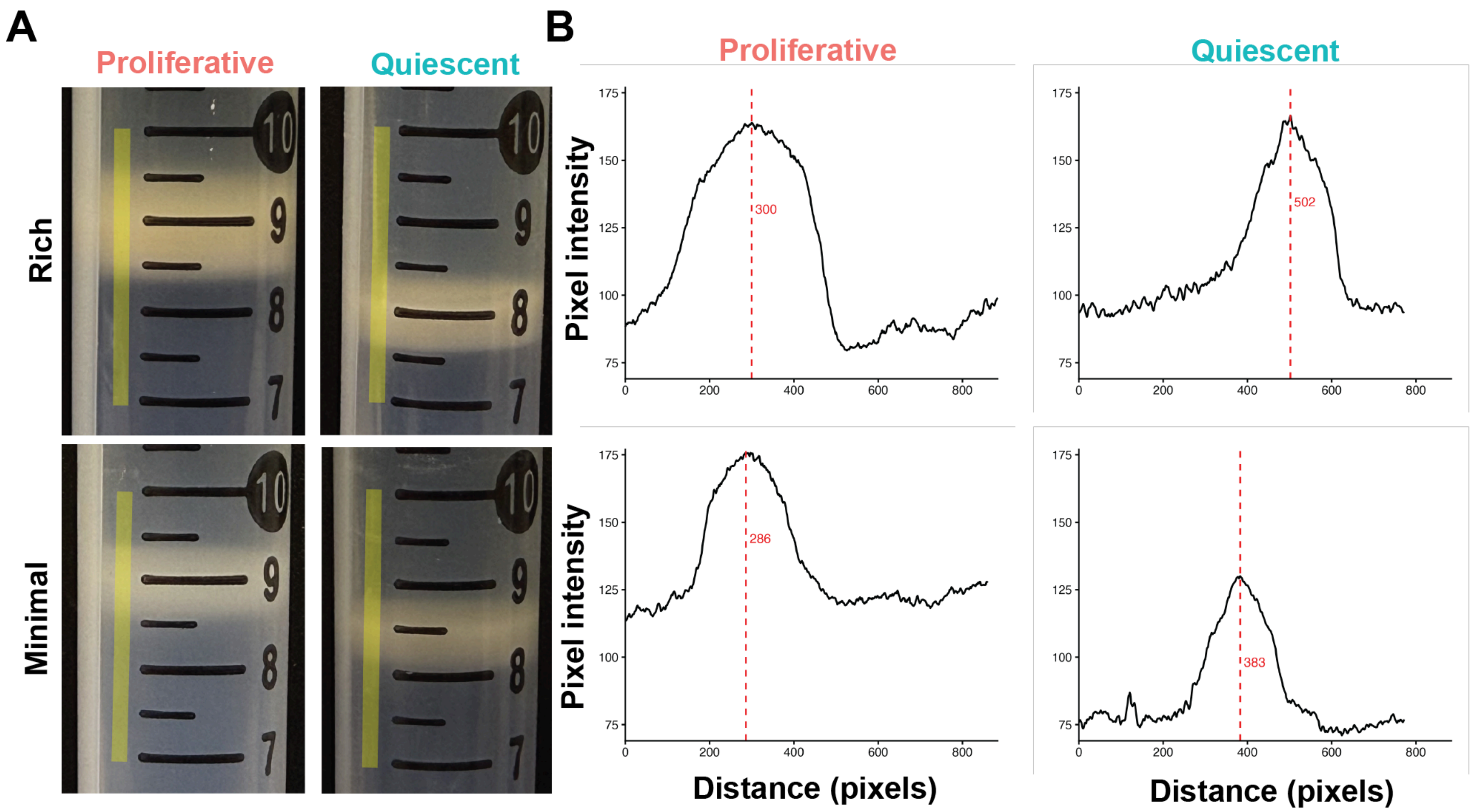

Supplementary Figure 3. Density fractionation of proliferative and quiescent cultures in rich and minimal media. **A)** Images of density fractionation indicating the region assessed for pixel quantification(yellow vertical line). **B)** Pixel intensity distribution across the analyzed region with maximum distance at which peak pixel intensity was achieved is indicated (red line with red text).

bioRxiv preprint doi: https://doi.org/10.64898/2026.02.27.708532; this version posted February 28, 2026. The copyright holder for this preprint (which was not certified by peer review) is the author/funder, who has granted bioRxiv a license to display the preprint in perpetuity. It is made available under a CC-BY 4.0 International license.

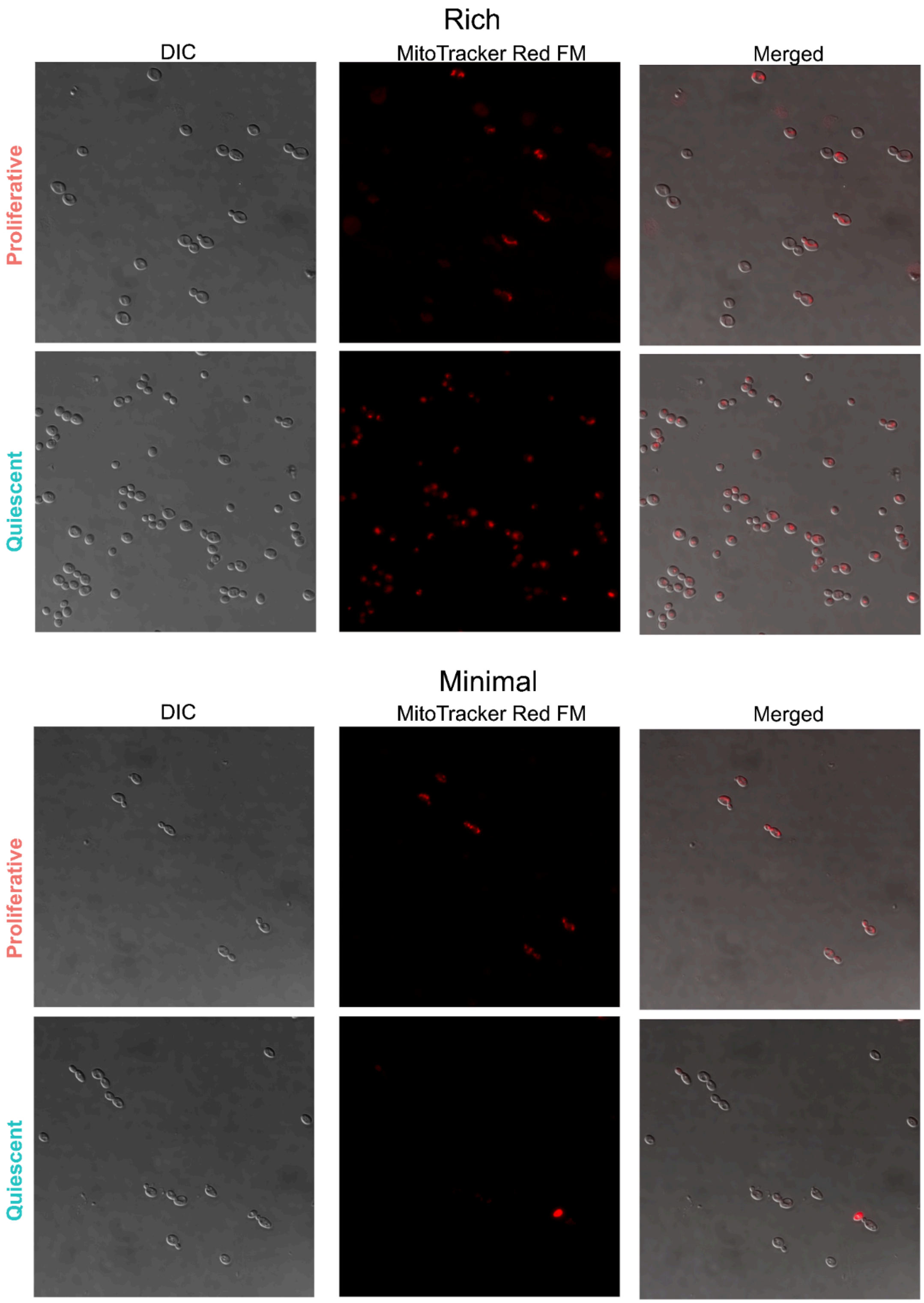

Supplementary Figure 4. Mitotracker Red FM staining of proliferative cultures in rich media. SC5314 was stained with 100 nM MT Red for 30 minutes at 37°C. MT Red fails to selectively stain mitochondria of cells from rich and minimal quiescent cultures.

bioRxiv preprint doi: https://doi.org/10.64898/2026.02.27.708532; this version posted February 28, 2026. The copyright holder for this preprint (which was not certified by peer review) is the author/funder, who has granted bioRxiv a license to display the preprint in perpetuity. It is made available under a CC-BY 4.0 International license.

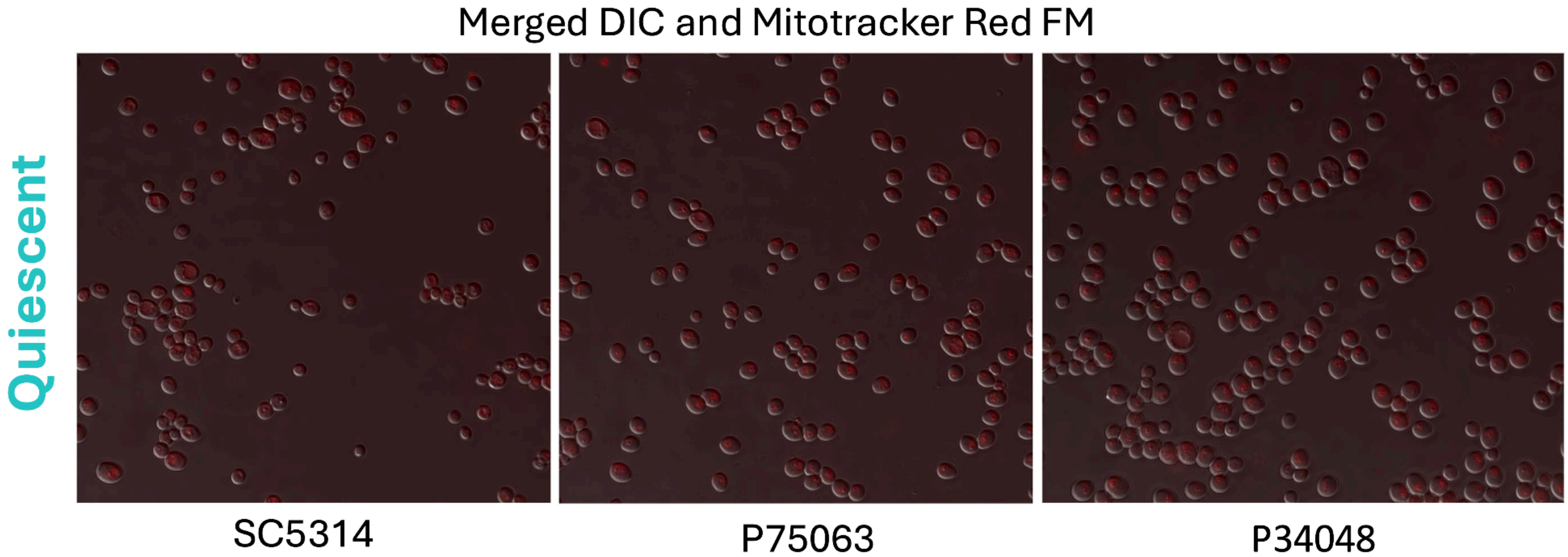

Supplementary Figure 5. Mitotracker Red FM fails to selectively stain mitochondria in quiescent cultures of the lab strain SC5314 and two clinical isolates. Quiescent cultures of SC5314, P75063, and P34048 grown in rich media, incubated with Mitotracker Red FM, and imaged.

bioRxiv preprint doi: https://doi.org/10.64898/2026.02.27.708532; this version posted February 28, 2026. The copyright holder for this preprint (which was not certified by peer review) is the author/funder, who has granted bioRxiv a license to display the preprint in perpetuity. It is made available under a CC-BY 4.0 International license.

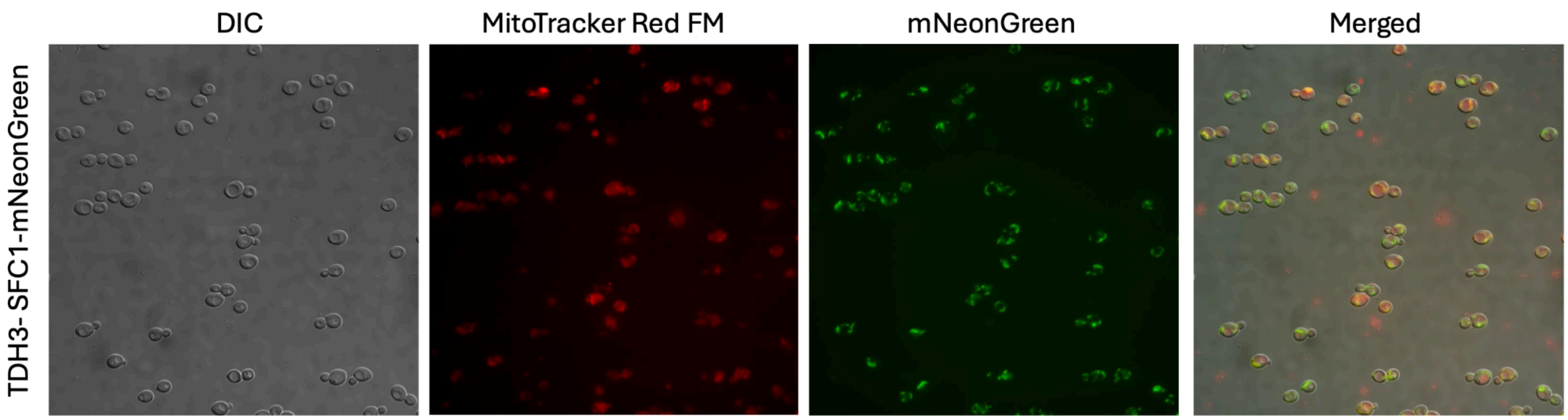

Supplementary Figure 6. Colocalization of mNeonGreen-SFC1 and Mitotracker Red FM. Proliferative cells containing the mNeonGreen-SFC1 protein fusion were grown in rich media and stained with Mitotracker Red FM.

bioRxiv preprint doi: https://doi.org/10.64898/2026.02.27.708532; this version posted February 28, 2026. The copyright holder for this preprint (which was not certified by peer review) is the author/funder, who has granted bioRxiv a license to display the preprint in perpetuity. It is made available under a CC-BY 4.0 International license.

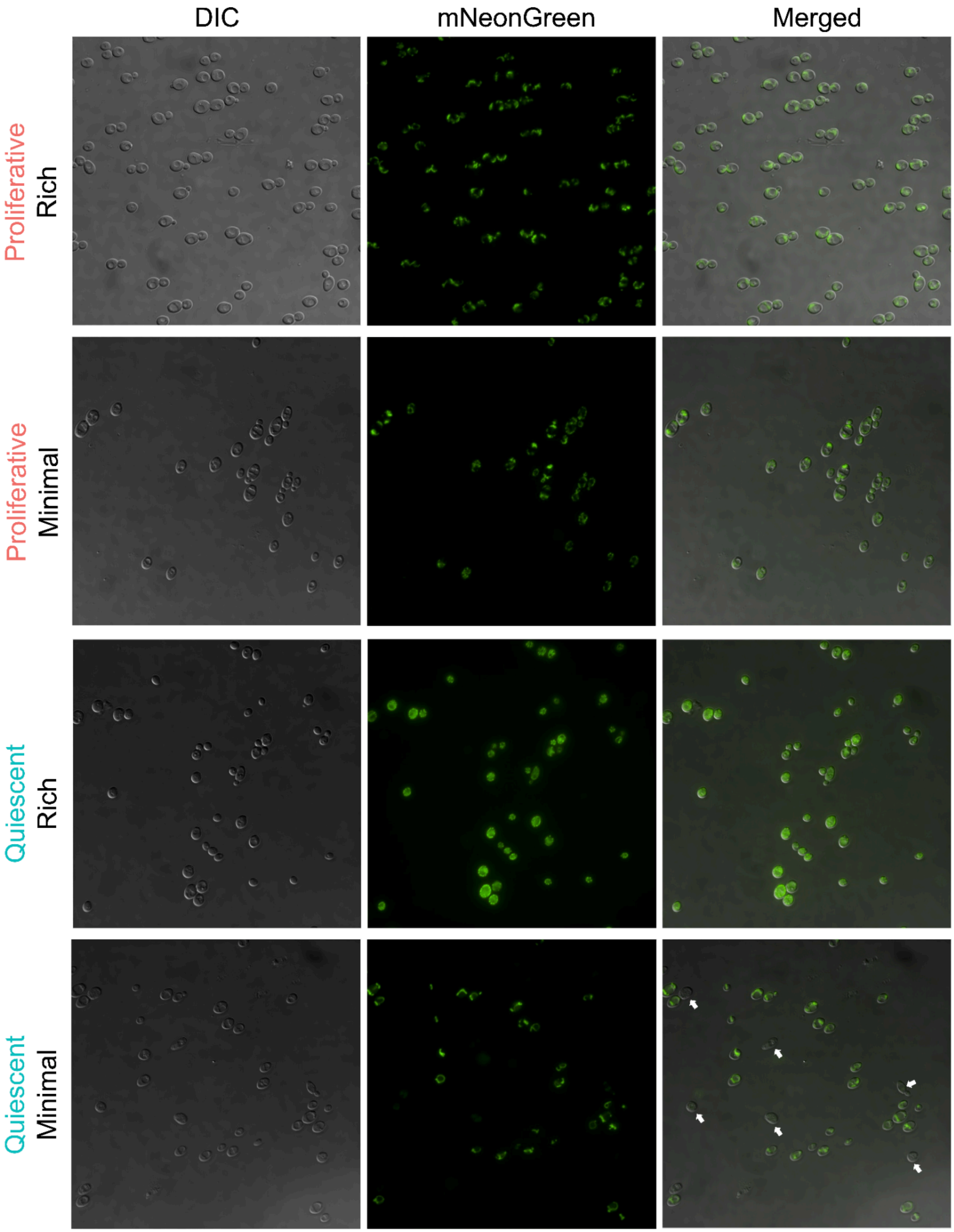

Supplementary Figure 7. Imaging of mitochondria using mNeonGreen-SFC1 in quiescent and proliferative cells grown in rich and minimal media. White arrows indicate cells that lack any mNeonGreen-SFC1 signal.

bioRxiv preprint doi: https://doi.org/10.64898/2026.02.27.708532; this version posted February 28, 2026. The copyright holder for this preprint (which was not certified by peer review) is the author/funder, who has granted bioRxiv a license to display the preprint in perpetuity. It is made available under a CC-BY 4.0 International license.

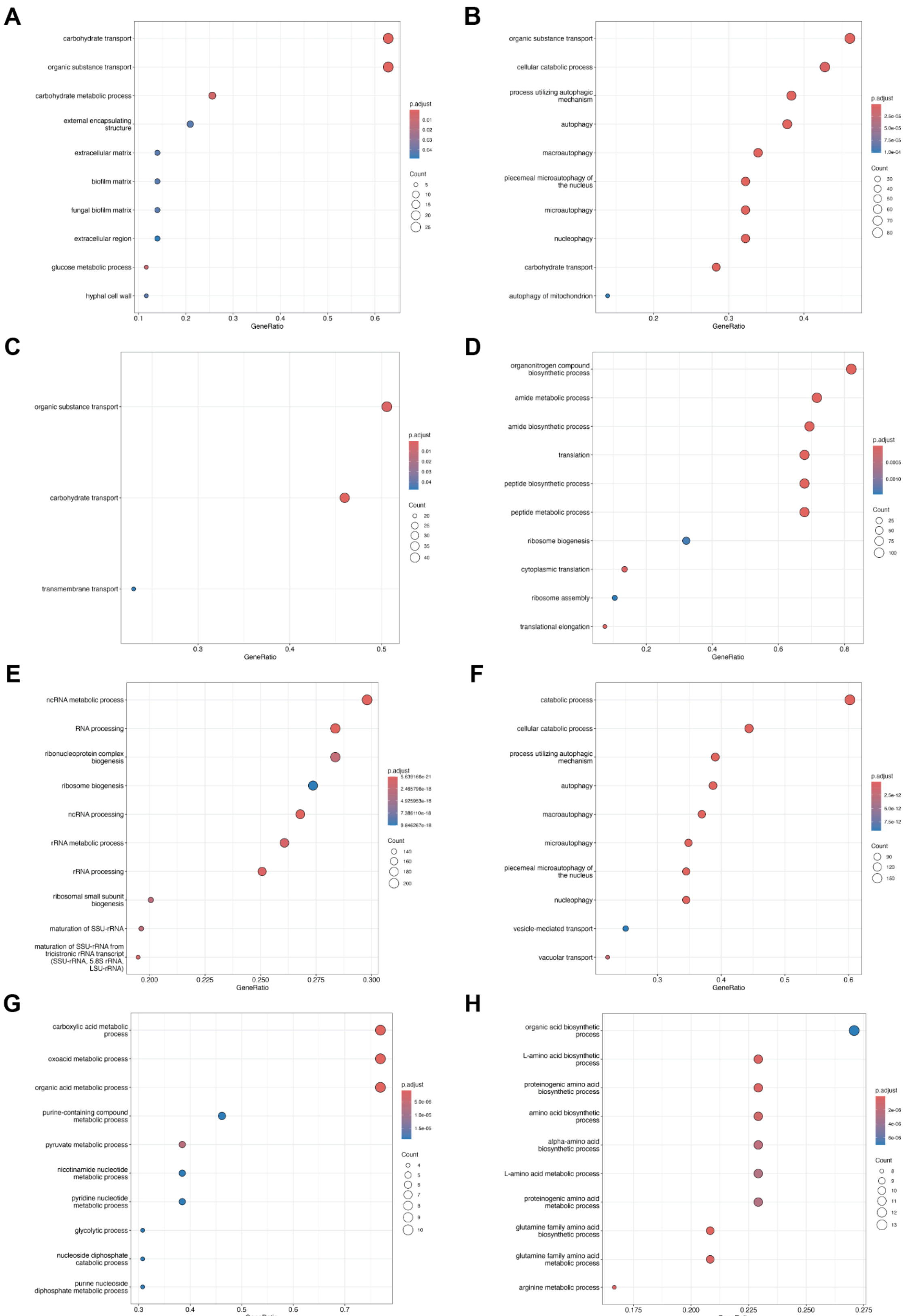

Supplementary Figure 8. Overrepresentation analysis of differentially expressed gene clusters defined in Figure 2. Dotplot showing over–represented GO term descriptions for genes in **A)** cluster 1, **B)** cluster 2, **C)** cluster 3, **D)** cluster 4, **E)** cluster 5, **F)** cluster 6, **G)** cluster 7, and **H)** cluster 8.

bioRxiv preprint doi: https://doi.org/10.64898/2026.02.27.708532; this version posted February 28, 2026. The copyright holder for this preprint (which was not certified by peer review) is the author/funder, who has granted bioRxiv a license to display the preprint in perpetuity. It is made available under a CC-BY 4.0 International license.

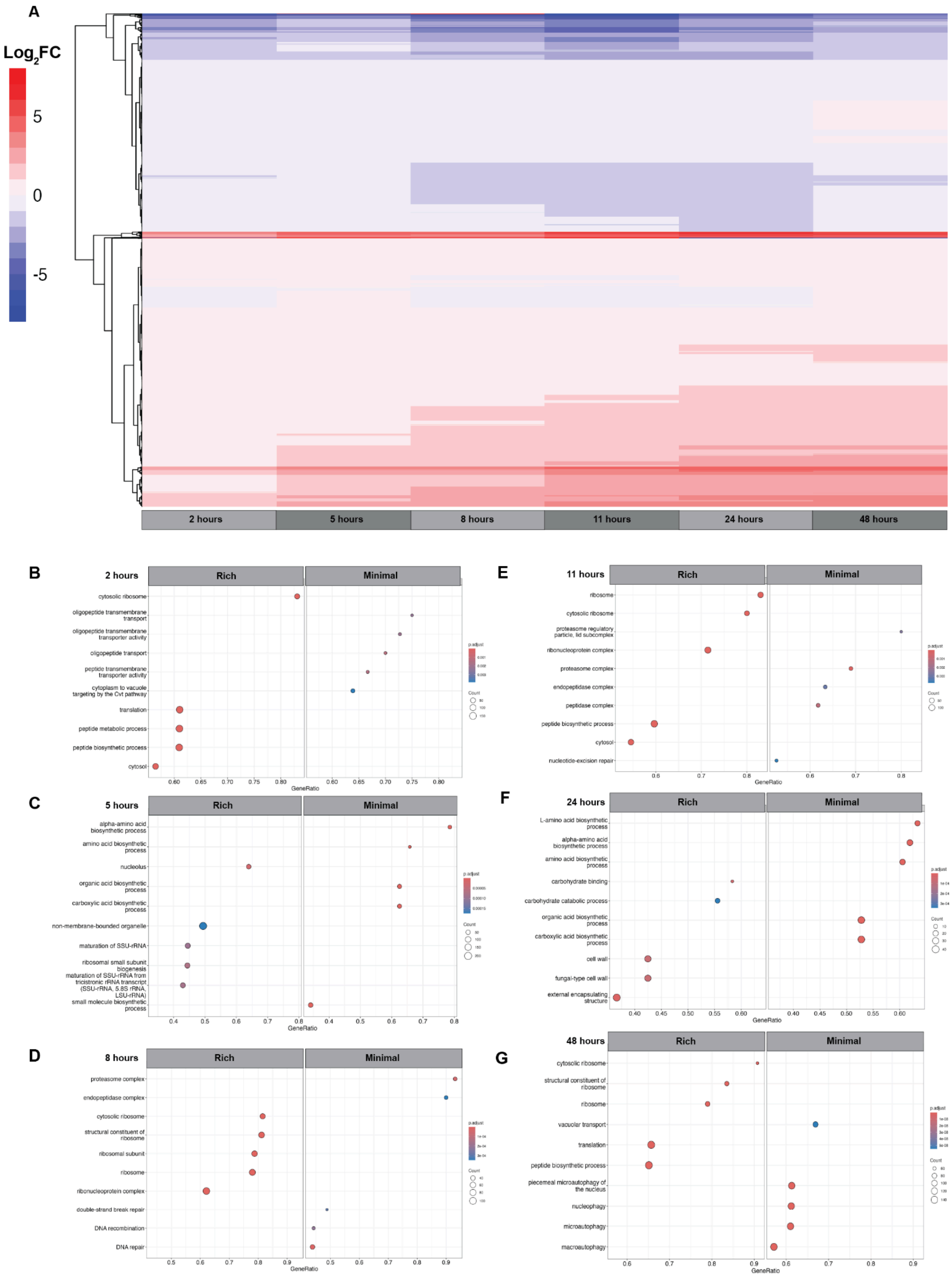

Supplementary Figure 9. Differential gene expression analysis of media conditions . A) Heatmap of the relative $\log_2$-transformed fold changes of gene expression in rich media compared to minimal media at each time point. The data were clustered using hierarchical clustering. Gene Set Enrichment Analysis (GSEA) was used to find enriched GO terms at **B)** 2 hours post-inoculation, **C)** 5 hours post-inoculation, **D)** 8 hours post-inoculation, **E)** 11 hours post-inoculation, **F)** 24 hours post-inoculation, and **G)** 48 hours post-inoculation. Only significant GO terms (adjusted p-value < 0.05) are shown.

bioRxiv preprint doi: https://doi.org/10.64898/2026.02.27.708532; this version posted February 28, 2026. The copyright holder for this preprint (which was not certified by peer review) is the author/funder, who has granted bioRxiv a license to display the preprint in perpetuity. It is made available under a CC-BY 4.0 International license.

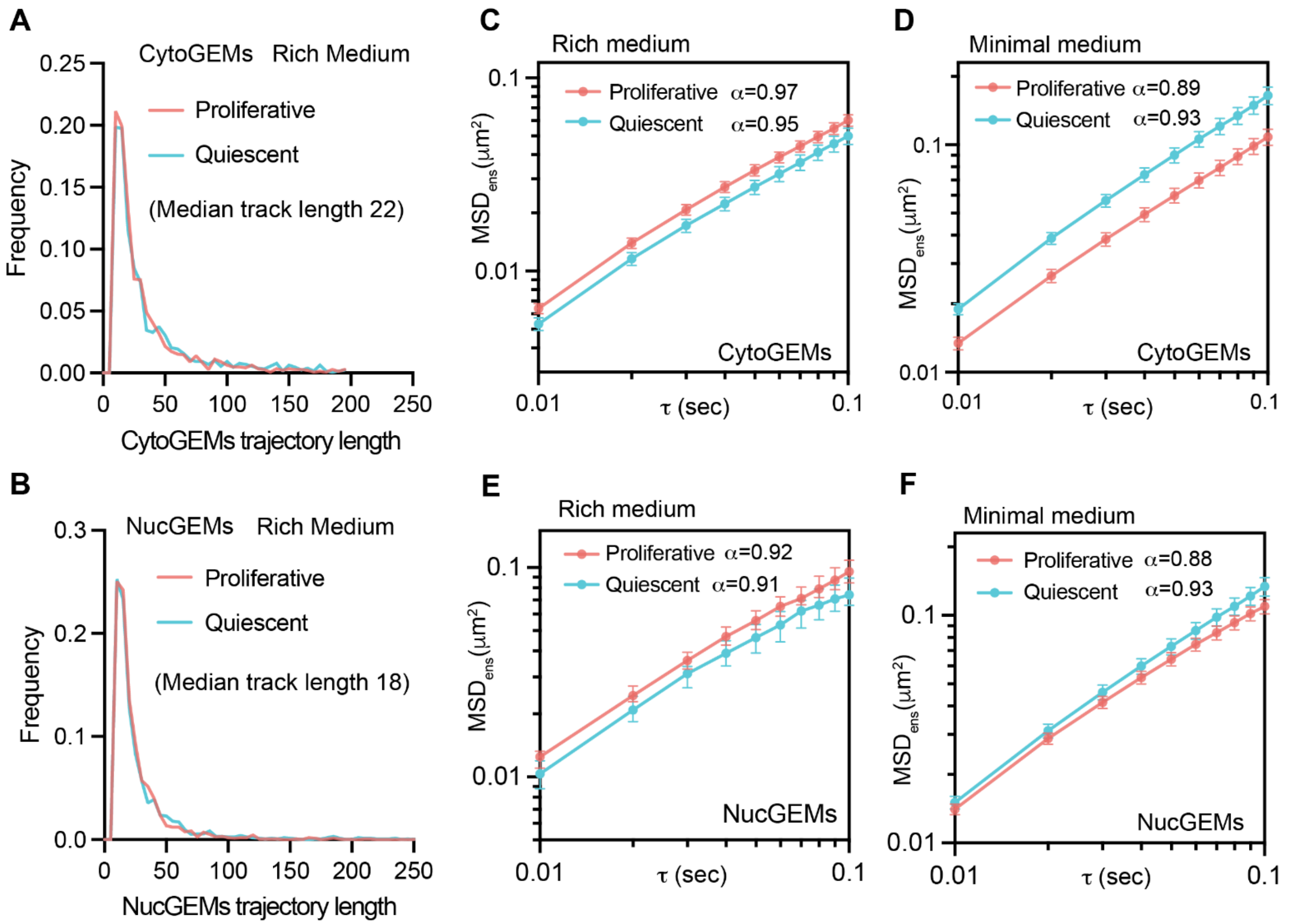

Supplementary Figure 10. Molecular Crowding Profiles of SC5314 Nucleus and Cytoplasm in Rich and Minimal Media. A) The frequency distribution of cytoplasmic 40nm-GEMs trajectory length in rich media. Proliferative: n = 1434 trajectories; quiescent: n = 1280 trajectories. **B)** The frequency distribution of nuclear 40nm-GEMs trajectory length in rich media. Proliferative: n = 1570 trajectories; quiescent: n = 1567 trajectories. **C)** Ensemble-averaged mean-squared displacement (MSD) versus time delay (τ), $\log_{10}$ scale, for cytoplasmic GEMs in cells growing in rich medium. Proliferative: n = 111 cells; quiescent: n = 80 cells. **D)** MSD vs. τ plot for cytoplasmic GEMs in cells growing in minimal medium. Proliferative: 114 cells; quiescent: 58 cells. **E)** MSD vs. τ for nuclear GEMs in cells growing in rich medium. Proliferative: n = 139 cells; quiescent: n = 150 cells. **F)** MSD vs. τ for cytoplasmic GEMs in cells growing in rich medium. Proliferative: n = 140 cells; quiescent: n = 136 cells. For the MSD quantifications, a linear model was fit to determine the anomalous exponent α values for 40nm-GEMs in *C. albicans* cells. Median ± 95% confidence interval.

bioRxiv preprint doi: https://doi.org/10.64898/2026.02.27.708532; this version posted February 28, 2026. The copyright holder for this preprint (which was not certified by peer review) is the author/funder, who has granted bioRxiv a license to display the preprint in perpetuity. It is made available under a CC-BY 4.0 International license.

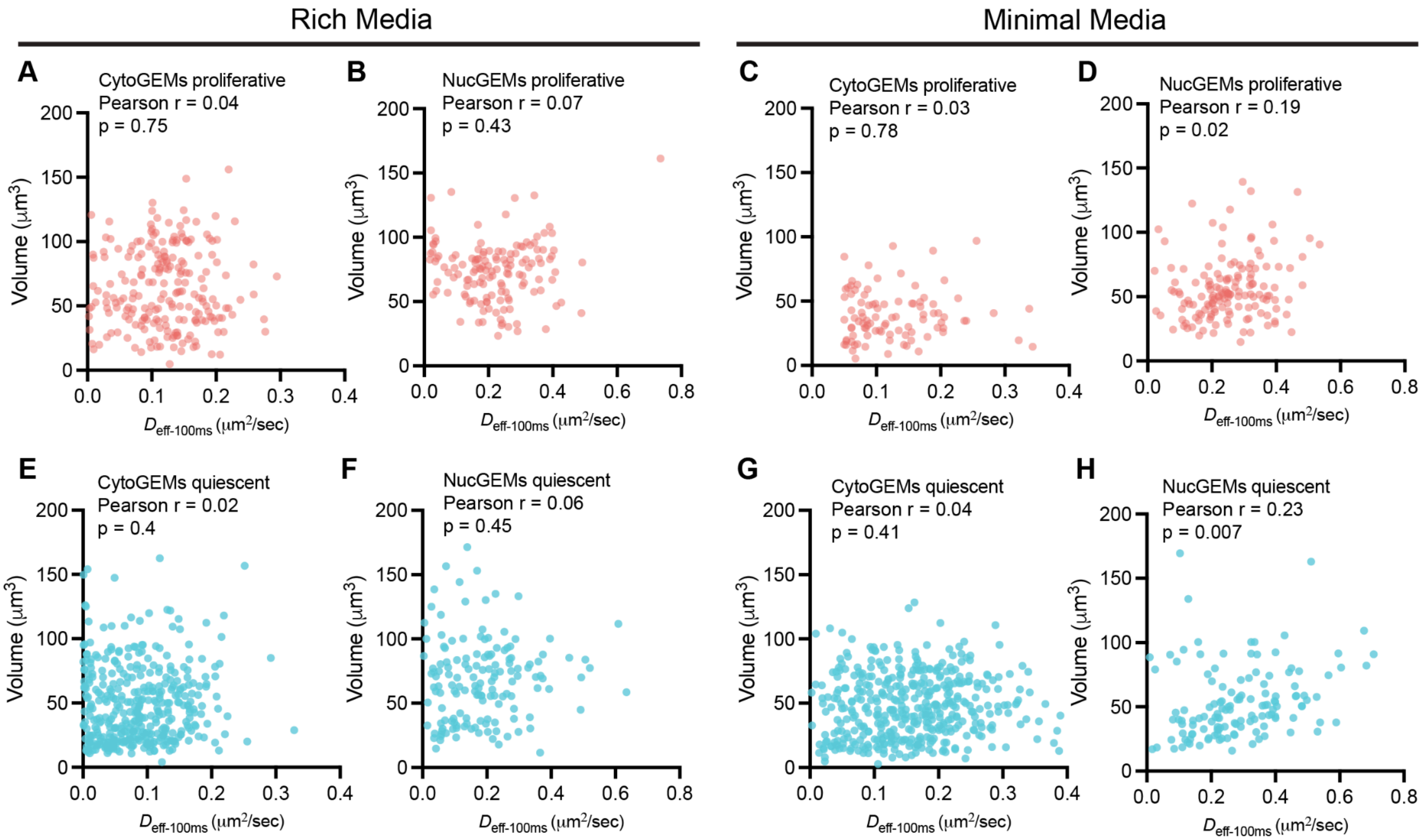

Supplementary Figure 11. Relationship between cell volume and 40nm-GEMs effective diffusion coefficients. A) Cell volume to 40nm-GEM diffusion coefficient scatterplot for GEMs located in the cytoplasm of proliferative cells grown in rich media. **B)** Cell volume to 40nm-GEM diffusion coefficient scatterplot for GEMs located in the nucleus of proliferative cells grown in rich media. **C)** Cell volume to 40nm-GEM diffusion coefficient scatterplot for GEMs located in the cytoplasm of proliferative cells grown in minimal media. **D)** Cell volume to 40nm-GEM diffusion coefficient scatterplot for GEMs located in the cytoplasm of proliferative cells grown in minimal media. **E)** Cell volume to 40nm-GEM diffusion coefficient scatterplot for GEMs located in the cytoplasm of quiescent cells grown in rich media. **F)** Cell volume to 40nm-GEM diffusion coefficient scatterplot for GEMs located in the nucleus of quiescent cells grown in rich media. **G)** Cell volume to 40nm-GEM diffusion coefficient scatterplot for GEMs located in the cytoplasm of quiescent cells grown in minimal media. **H)** Cell volume to 40nm-GEM diffusion coefficient scatterplot for GEMs located in the nucleus of quiescent cells grown in minimal media. Pearson correlation r is calculated between diffusion coefficient (x-axis) and cell volume (y-axis) (Each dot represents one cell, p value was calculated by two tailed t test).

bioRxiv preprint doi: https://doi.org/10.64898/2026.02.27.708532; this version posted February 28, 2026. The copyright holder for this preprint (which was not certified by peer review) is the author/funder, who has granted bioRxiv a license to display the preprint in perpetuity. It is made available under a CC-BY 4.0 International license.

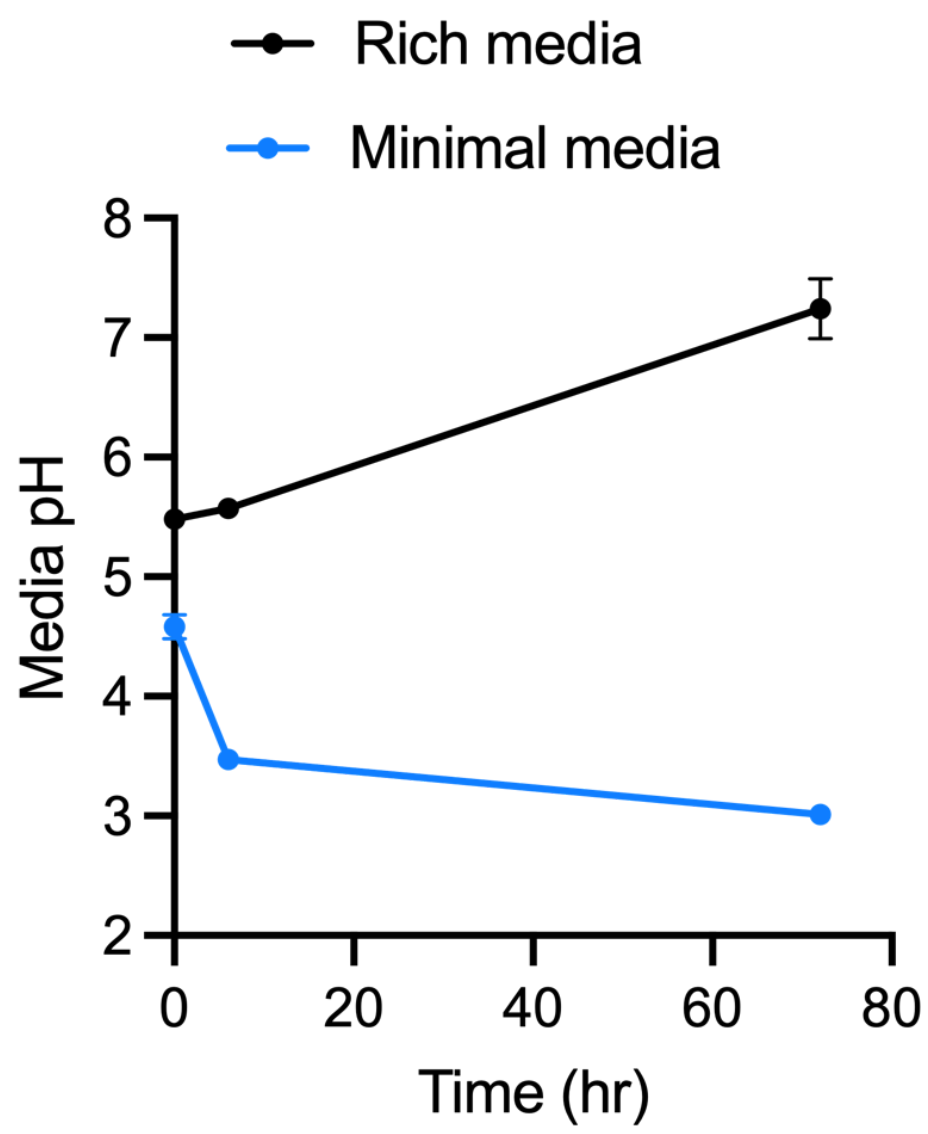

Supplementary Figure 12. Culture media pH was measured at the indicated time points after C. albicans growth. Three culture tubes were used in two types of media (mean ± standard deviation)

bioRxiv preprint doi: https://doi.org/10.64898/2026.02.27.708532; this version posted February 28, 2026. The copyright holder for this preprint (which was not certified by peer review) is the author/funder, who has granted bioRxiv a license to display the preprint in perpetuity. It is made available under a CC-BY 4.0 International license.

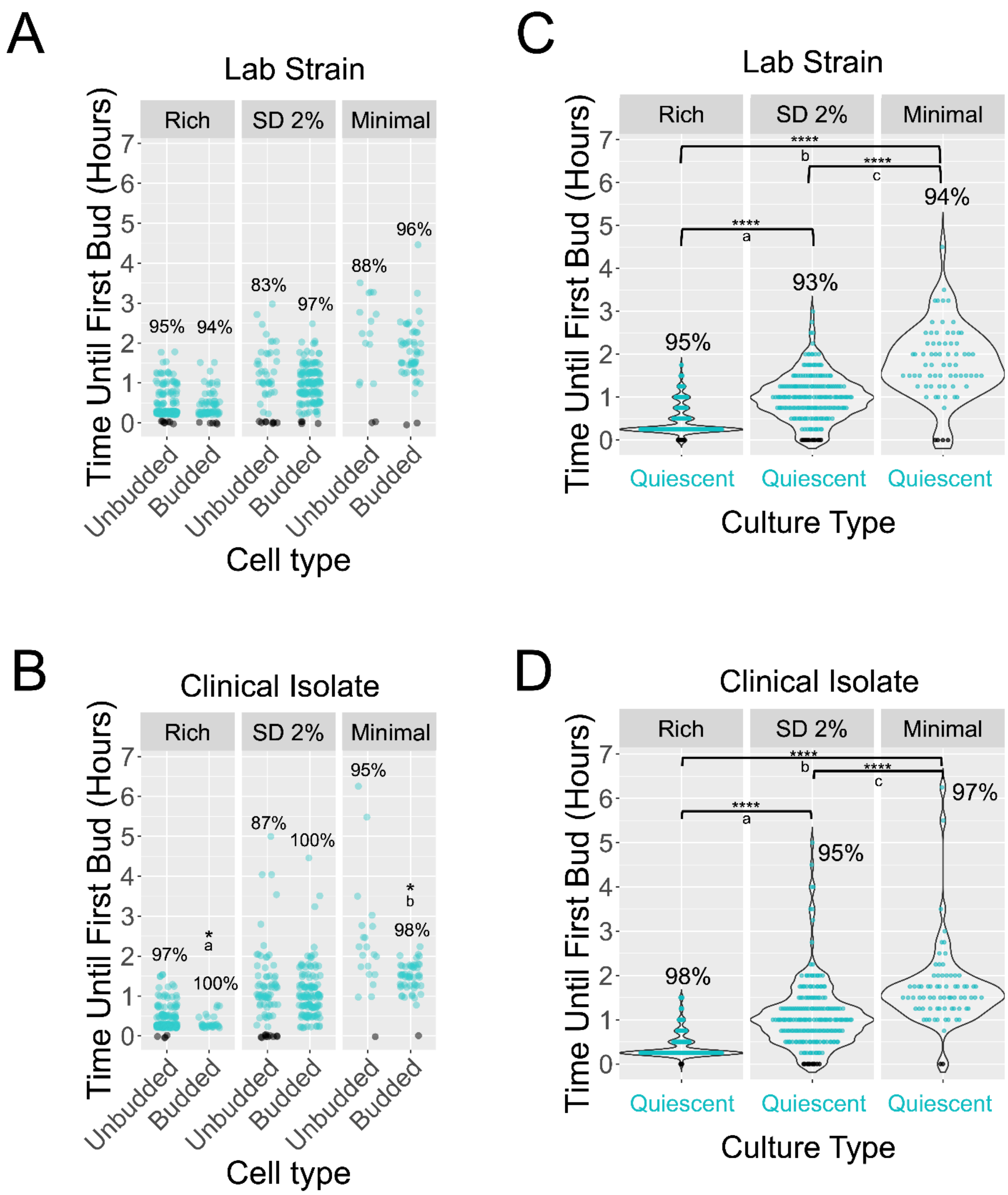

Supplementary Figure 13. Time to bud emergence and budding status of the lab strain and a clinical isolate. A) Comparison of unbudded versus budded time until first bud emergence in quiescent cultures of the lab strain SC5314 in rich, SD 2% glucose, and minimal medium. The percentage of cells that divided within the 9-hour timeframe is noted above each bud category. **B)** Comparison of unbudded versus budded time until first bud emergence in quiescent cultures of the clinical isolate P34048 in rich, SD 2% glucose, and minimal medium. The percentage of cells that divided within the 9-hour timeframe is noted above each bud category. p-values for: a) 0.0437; b) 0.0105. **C)** Time until first bud emergence from quiescent cultures of the lab strain SC5314 grown in three types of media (rich and minimal media show the same data from Figure 4B). p-values for a) 2.40E-29; b) 6.51E-22; c) 2.08E-11 **D)** Time until first bud emergence from quiescent cultures of the clinical isolate P34048 grown in three types of media (rich and minimal media show the same data from Figure 4B). p-values for a) 4.08E-28; b) 6.80E-20; c) 1.22E-06. SC5314 rich quiescent, n = 243; SC5314 SD 2% quiescent, n = 199; SC5314 minimal quiescent, n = 70; P34048 rich quiescent, n = 208; P34048 SD 2% quiescent, n = 205; P34048 minimal quiescent, n = 74. p-values for significant differences between conditions or budding status are denoted with asterisks. The p-value was determined using Student's t-test, *p < 0.05, **p < 0.01, ***p < 0.001, ****p < 0.0001).

bioRxiv preprint doi: https://doi.org/10.64898/2026.02.27.708532; this version posted February 28, 2026. The copyright holder for this preprint (which was not certified by peer review) is the author/funder, who has granted bioRxiv a license to display the preprint in perpetuity. It is made available under a CC-BY 4.0 International license.

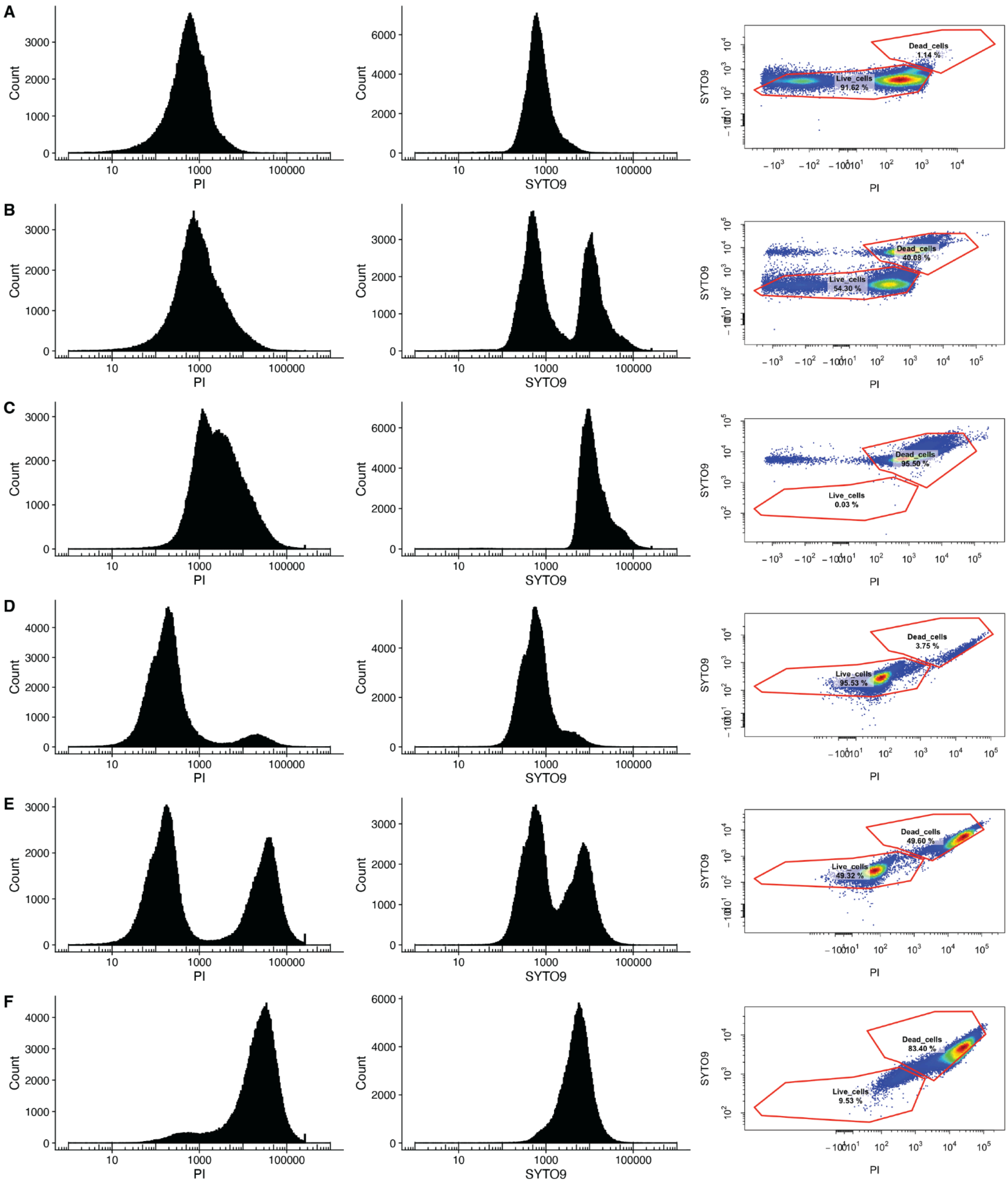

Supplementary Figure 14. Validation of PI/SYTO9 staining and flow cytometry to measure viability of proliferative and quiescent cells. In each panel the distribution of PI (left panel), SYTO9 (middle panel) and the scatter plot of PI versus SYTO9 used to define flow cytometry gates is displayed for **A)** untreated proliferative cells grown (100% viability expected), **B)** a 50:50 mixture of untreated cells and cells exposed to 68℃ for 1 hour (50% viability expected) **C)** cells exposed to 68℃ for 1 hour (100% inviability expected) **D)** untreated quiescent cells (100% viability expected), **E)** a 50:50 mixture of untreated quiescent cells and quiescent cells exposed to 68℃ for 1 hour (50% viability expected), and **F)** quiescent cells exposed to 68℃ for 1 hour (100% inviability expected). For the experiment, all cells were grown in rich media.

bioRxiv preprint doi: https://doi.org/10.64898/2026.02.27.708532; this version posted February 28, 2026. The copyright holder for this preprint (which was not certified by peer review) is the author/funder, who has granted bioRxiv a license to display the preprint in perpetuity. It is made available under a CC-BY 4.0 International license.

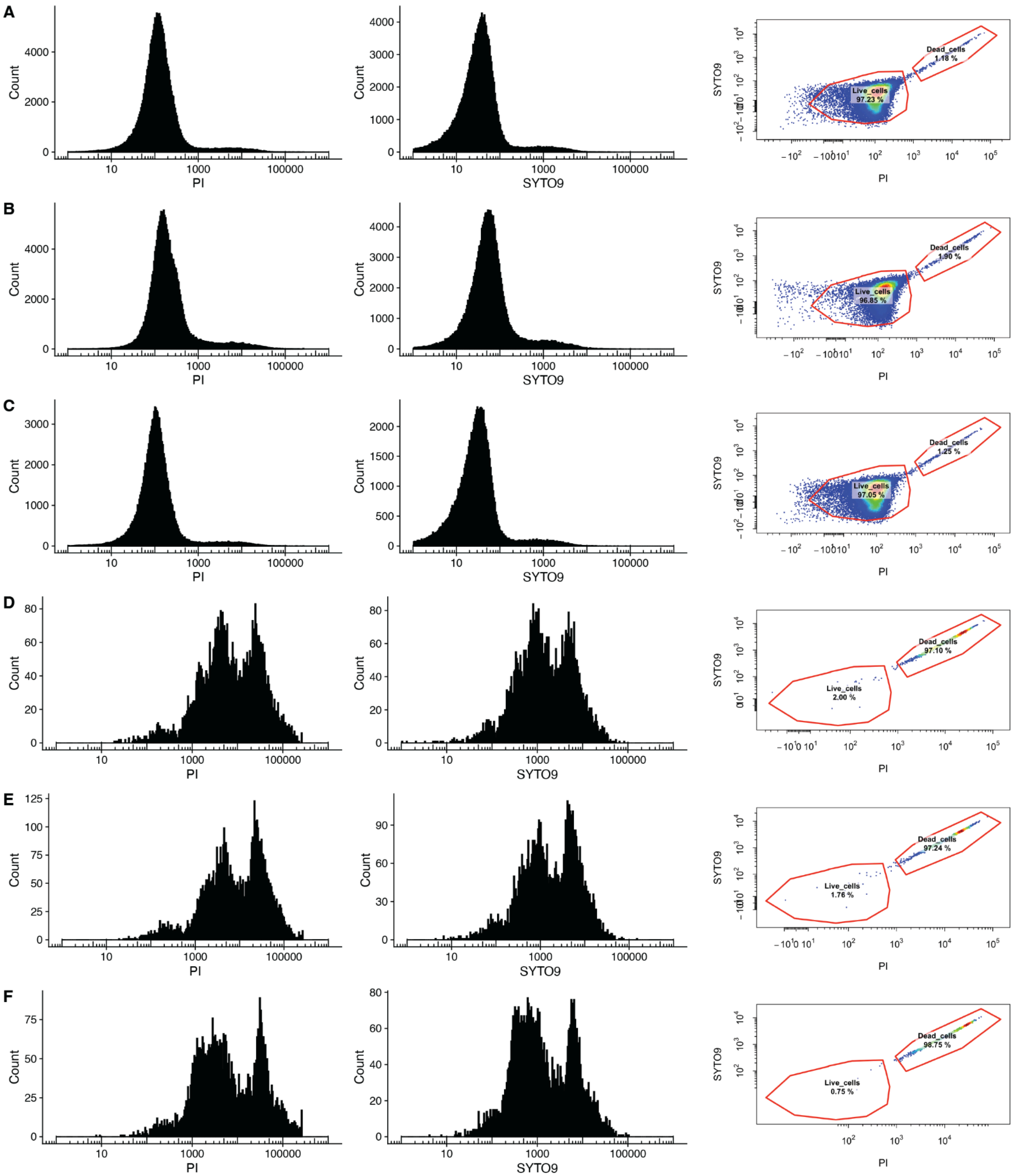

Supplementary Figure 15. Cell viability quantification to validate accuracy of PI/SYTO9 staining quantification with caspofungin exposed proliferative cells. A) PI and SYTO9 distribution functions as well as the gated PI/SYTO9 2D quantification of untreated proliferative cells grown in rich media. **B)** PI and SYTO9 distribution functions as well as the gated PI/SYTO9 2D quantification of proliferative cells exposed to 0.001ug/mL caspofungin grown in rich media. **C)** PI and SYTO9 distribution functions as well as the gated PI/SYTO9 2D quantification of cells exposed to 0.01ug/mL caspofungin grown in rich media. **D)** PI and SYTO9 distribution functions as well as the gated PI/SYTO9 2D quantification of cells exposed to 0.1ug/mL caspofungin grown in rich media. **E)** PI and SYTO9 distribution functions as well as the gated PI/SYTO9 2D quantification of cells exposed to 1ug/mL caspofungin grown in rich media. **F)** PI and SYTO9 distribution functions as well as the gated PI/SYTO9 2D quantification of cells exposed to 10ug/mL caspofungin grown in rich media.

bioRxiv preprint doi: https://doi.org/10.64898/2026.02.27.708532; this version posted February 28, 2026. The copyright holder for this preprint (which was not certified by peer review) is the author/funder, who has granted bioRxiv a license to display the preprint in perpetuity. It is made available under a CC-BY 4.0 International license.

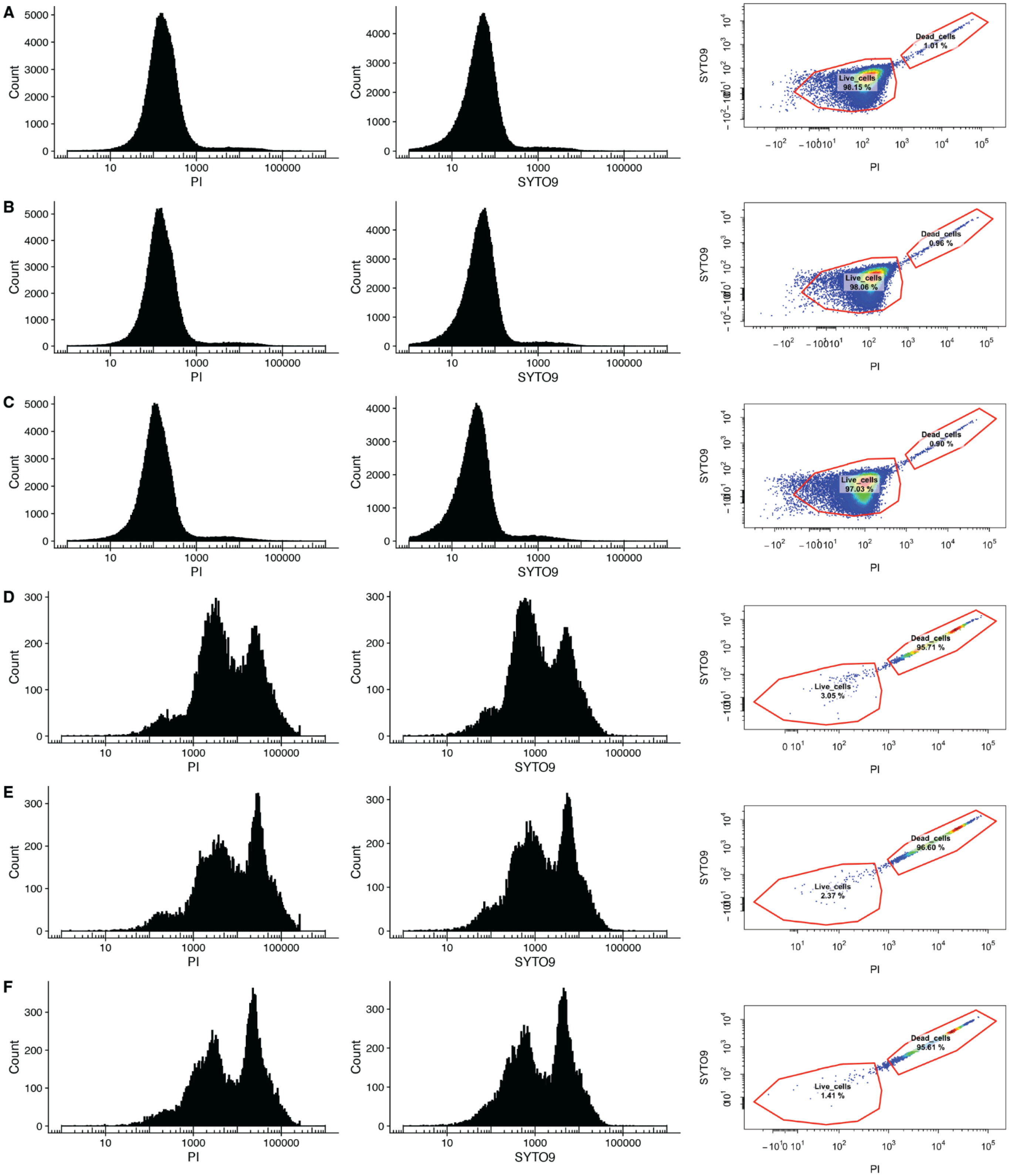

Supplementary Figure 16. Cell viability quantification to validate accuracy of PI/SYTO9 staining quantification with micafungin exposed proliferative cells. A) PI and SYTO9 distribution functions as well as the gated PI/SYTO9 2D quantification of untreated proliferative cells grown in rich media. **B)** PI and SYTO9 distribution functions as well as the gated PI/SYTO9 2D quantification of proliferative cells exposed to 0.001ug/mL micafungin grown in rich media. **C)** PI and SYTO9 distribution functions as well as the gated PI/SYTO9 2D quantification of cells exposed to 0.01ug/mL micafungin grown in rich media. **D)** PI and SYTO9 distribution functions as well as the gated PI/SYTO9 2D quantification of cells exposed to 0.1ug/mL micafungin grown in rich media. **E)** PI and SYTO9 distribution functions as well as the gated PI/SYTO9 2D quantification of cells exposed to 1ug/mL micafungin grown in rich media. **F)** PI and SYTO9 distribution functions as well as the gated PI/SYTO9 2D quantification of cells exposed to 10ug/mL micafungin grown in rich media.

bioRxiv preprint doi: https://doi.org/10.64898/2026.02.27.708532; this version posted February 28, 2026. The copyright holder for this preprint (which was not certified by peer review) is the author/funder, who has granted bioRxiv a license to display the preprint in perpetuity. It is made available under a CC-BY 4.0 International license.

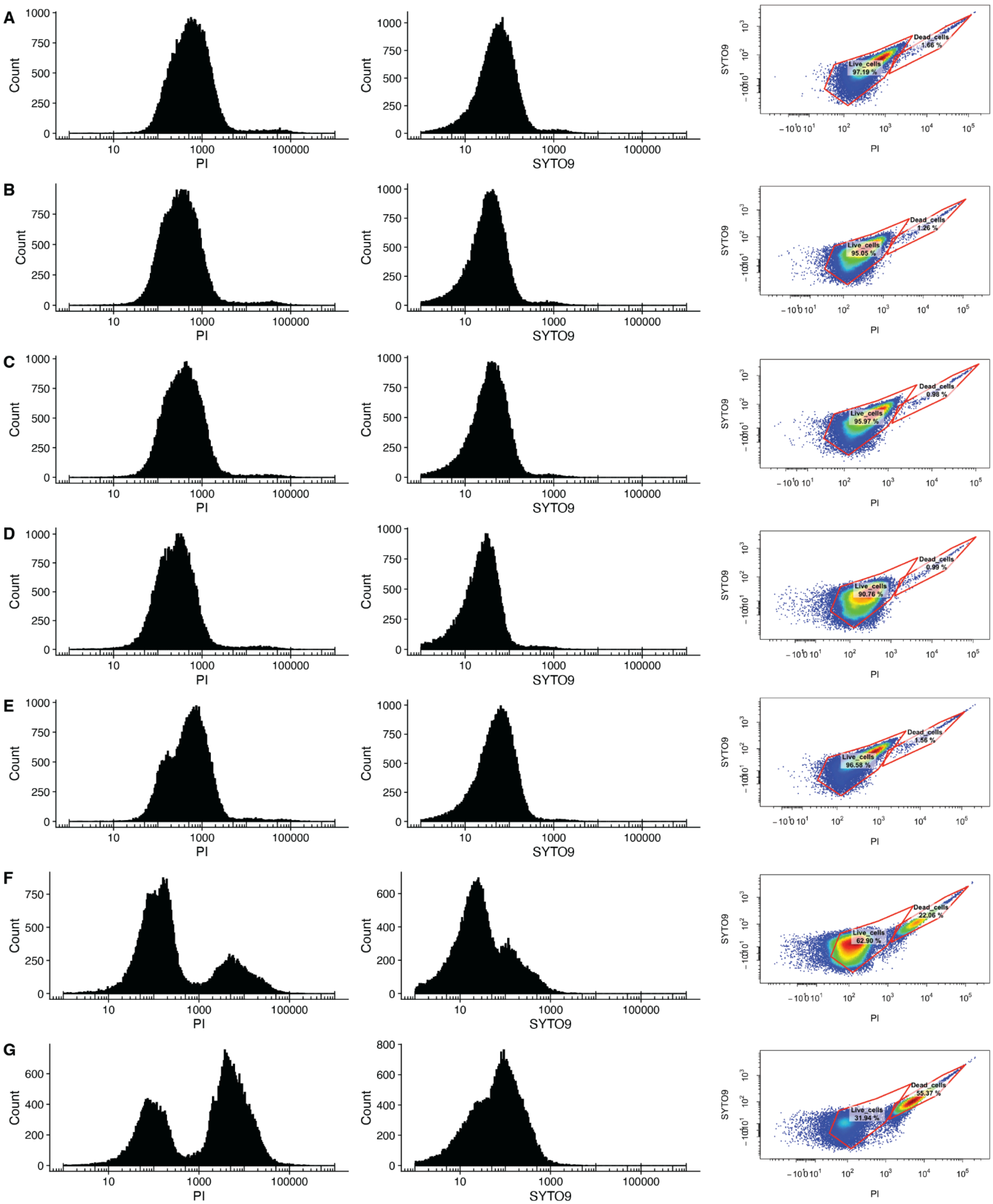

Supplementary Figure 17. Cell viability quantification to validate accuracy of PI/SYTO9 staining quantification with amphotericin B exposed proliferative cells. A) PI and SYTO9 distribution functions as well as the gated PI/SYTO9 2D quantification of untreated proliferative cells grown in rich media. **B)** PI and SYTO9 distribution functions as well as the gated PI/SYTO9 2D quantification of proliferative cells exposed to 0.001ug/mL amphotericin B grown in rich media. **C)** PI and SYTO9 distribution functions as well as the gated PI/SYTO9 2D quantification of cells exposed to 0.01ug/mL amphotericin B grown in rich media. **D)** PI and SYTO9 distribution functions as well as the gated PI/SYTO9 2D quantification of cells exposed to 0.1ug/mL amphotericin B grown in rich media. **E)** PI and SYTO9 distribution functions as well as the gated PI/SYTO9 2D quantification of cells exposed to 1ug/mL amphotericin B grown in rich media. **F)** PI and SYTO9 distribution functions as well as the gated PI/SYTO9 2D quantification of cells exposed to 10ug/mL amphotericin B grown in rich media. **G)** PI and SYTO9 distribution functions as well as the gated PI/SYTO9 2D quantification of cells exposed to 20ug/mL amphotericin B grown in rich media.

bioRxiv preprint doi: https://doi.org/10.64898/2026.02.27.708532; this version posted February 28, 2026. The copyright holder for this preprint (which was not certified by peer review) is the author/funder, who has granted bioRxiv a license to display the preprint in perpetuity. It is made available under a CC-BY 4.0 International license.

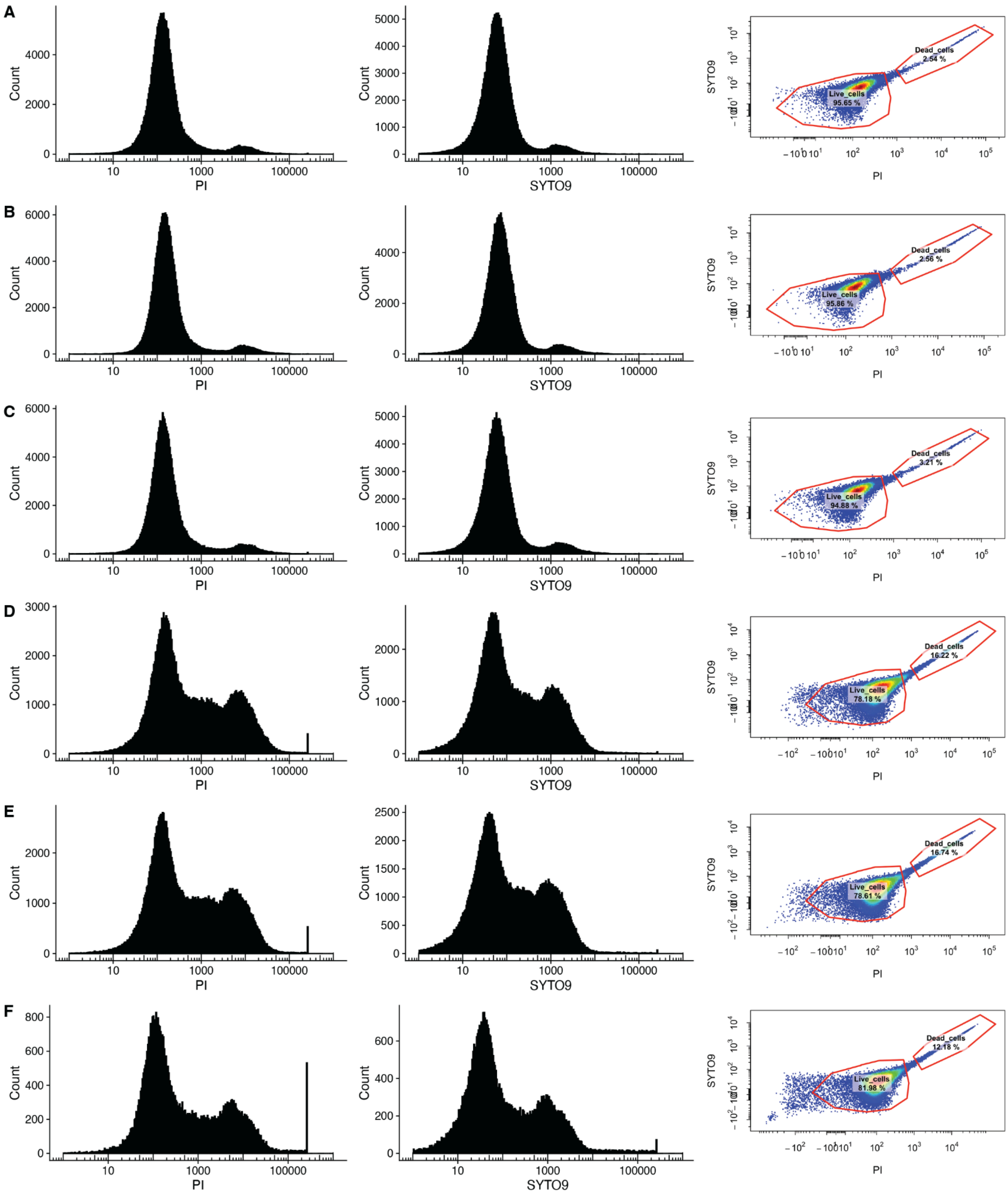

Supplementary Figure 18. Cell viability quantification to validate accuracy of PI/SYTO9 staining quantification with caspofungin exposed quiescent cells. A) PI and SYTO9 distribution functions as well as the gated PI/SYTO9 2D quantification of untreated quiescent cells grown in rich media. **B)** PI and SYTO9 distribution functions as well as the gated PI/SYTO9 2D quantification of quiescent cells exposed to 0.001ug/mL caspofungin grown in rich media. **C)** PI and SYTO9 distribution functions as well as the gated PI/SYTO9 2D quantification of quiescent cells exposed to 0.01ug/mL caspofungin grown in rich media. **D)** PI and SYTO9 distribution functions as well as the gated PI/SYTO9 2D quantification of quiescent cells exposed to 0.1ug/mL caspofungin grown in rich media. **E)** PI and SYTO9 distribution functions as well as the gated PI/SYTO9 2D quantification of quiescent cells exposed to 1ug/mL caspofungin grown in rich media. **F)** PI and SYTO9 distribution functions as well as the gated PI/SYTO9 2D quantification of quiescent cells exposed to 10ug/mL caspofungin grown in rich media.

bioRxiv preprint doi: https://doi.org/10.64898/2026.02.27.708532; this version posted February 28, 2026. The copyright holder for this preprint (which was not certified by peer review) is the author/funder, who has granted bioRxiv a license to display the preprint in perpetuity. It is made available under a CC-BY 4.0 International license.

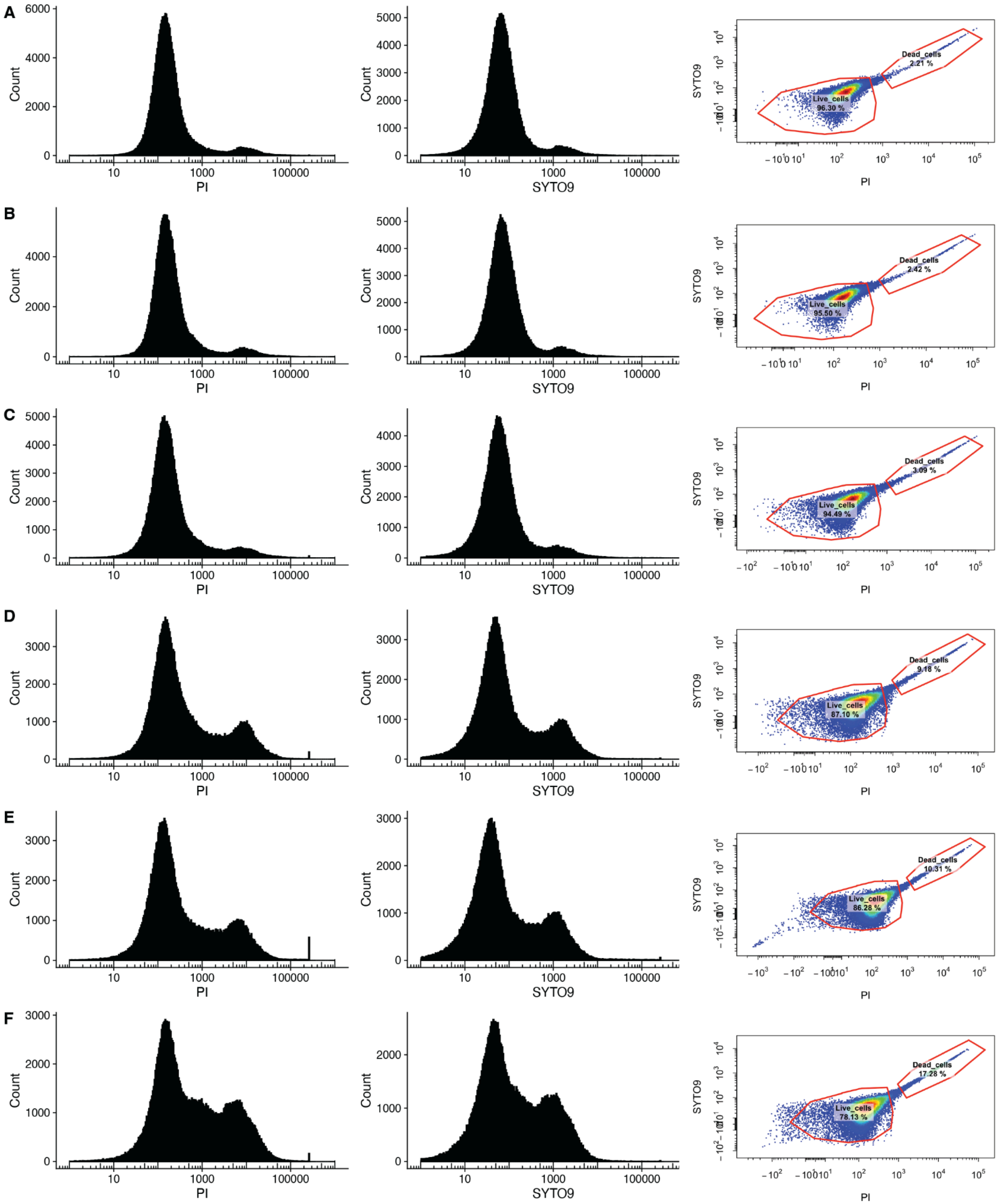

Supplementary Figure 19. Cell viability quantification to validate accuracy of PI/SYTO9 staining quantification with micafungin exposed quiescent cells. A) PI and SYTO9 distribution functions as well as the gated PI/SYTO9 2D quantification of untreated quiescent cells grown in rich media. **B)** PI and SYTO9 distribution functions as well as the gated PI/SYTO9 2D quantification of quiescent cells exposed to 0.001ug/mL micafungin grown in rich media. **C)** PI and SYTO9 distribution functions as well as the gated PI/SYTO9 2D quantification of quiescent cells exposed to 0.01ug/mL micafungin grown in rich media. **D)** PI and SYTO9 distribution functions as well as the gated PI/SYTO9 2D quantification of quiescent cells exposed to 0.1ug/mL micafungin grown in rich media. **E)** PI and SYTO9 distribution functions as well as the gated PI/SYTO9 2D quantification of quiescent cells exposed to 1ug/mL micafungin grown in rich media. **F)** PI and SYTO9 distribution functions as well as the gated PI/SYTO9 2D quantification of quiescent cells exposed to 10ug/mL micafungin grown in rich media.

bioRxiv preprint doi: https://doi.org/10.64898/2026.02.27.708532; this version posted February 28, 2026. The copyright holder for this preprint (which was not certified by peer review) is the author/funder, who has granted bioRxiv a license to display the preprint in perpetuity. It is made available under a CC-BY 4.0 International license.

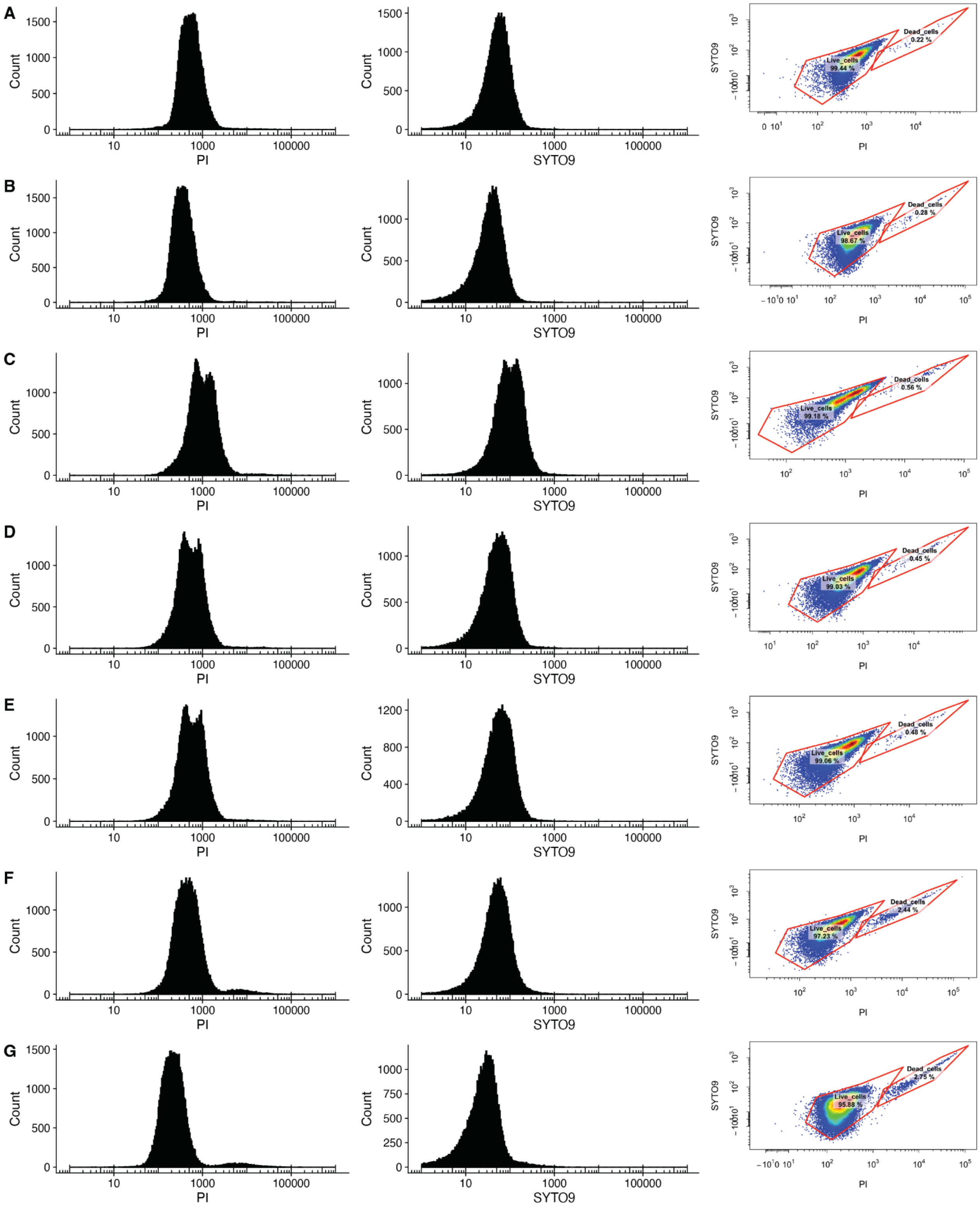

Supplementary Figure 20. Cell viability quantification to validate accuracy of PI/SYTO9 staining quantification with amphotericin B exposed quiescent cells. A) PI and SYTO9 distribution functions as well as the gated PI/SYTO9 2D quantification of untreated quiescent cells grown in rich media. **B)** PI and SYTO9 distribution functions as well as the gated PI/SYTO9 2D quantification of quiescent cells exposed to 0.001ug/mL amphotericin B grown in rich media. **C)** PI and SYTO9 distribution functions as well as the gated PI/SYTO9 2D quantification of quiescent cells exposed to 0.01ug/mL amphotericin B grown in rich media. **D)** PI and SYTO9 distribution functions as well as the gated PI/SYTO9 2D quantification of quiescent cells exposed to 0.1ug/mL amphotericin B grown in rich media. **E)** PI and SYTO9 distribution functions as well as the gated PI/SYTO9 2D quantification of quiescent cells exposed to 1ug/mL amphotericin B grown in rich media. **F)** PI and SYTO9 distribution functions as well as the gated PI/SYTO9 2D quantification of quiescent cells exposed to 10ug/mL amphotericin B grown in rich media. PI and SYTO9 distribution functions as well as the gated PI/SYTO9 2D quantification of cells exposed to 20ug/mL amphotericin B grown in rich media.
